# Evaluation of the Dose of African Swine Fever Virus Required to Establish Infection in Pigs Following Oral Uptake

**DOI:** 10.3390/pathogens14020119

**Published:** 2025-01-27

**Authors:** Ann Sofie Olesen, Christina Marie Lazov, Francesc Accensi, Camille Melissa Johnston, Thomas Bruun Rasmussen, Anette Bøtner, Louise Lohse, Graham J. Belsham

**Affiliations:** 1 Section for Veterinary Virology, Department of Virus & Microbiological Special Diagnostics, Statens Serum Institut, DK-2300 Copenhagen, Denmark; chlv@ssi.dk (C.M.L.); camj@ssi.dk (C.M.J.); tbru@ssi.dk (T.B.R.); lolo@ssi.dk (L.L.); 2 Section for Veterinary Clinical Microbiology, Department of Veterinary and Animal Sciences, University of Copenhagen, DK-1870 Frederiksberg C, Denmark; anettebotner@outlook.com (A.B.); grbe@sund.ku.dk (G.J.B.); 3 Research Combined Unit IRTA-UAB in Animal Health, Research Center in Animal Health (Centre de Recerca en Sanitat Animal CReSA), Campus of Autonomous University of Barcelona (UAB), 08193 Bellaterra, Spain; francesc.accensi@uab.cat; 4 Departament de Sanitat i Anatomia Animals, Facultat de Veterinària, Campus of Autonomous University of Barcelona UAB, 08193 Bellaterra, Spain; 5 WOAH Collaborating Centre for the Research and Control of Emerging and Re-Emerging Swine Diseases in Europe (IRTA-CReSA), 08193 Bellaterra, Spain

**Keywords:** ASFV, dosing study, feeding, transmission, oral uptake

## Abstract

African swine fever virus (ASFV) is known to be very stable within a protein-rich environment and indirect virus transmission can be mediated via oral uptake of different materials. However, experimental studies in pigs have shown that infection by ASFV via the oral route can be difficult to establish. Currently, there is a lack of studies using strict oral inoculations of pigs with different doses of ASFV. Therefore, we aimed to determine the dose of a European genotype II ASFV that is required to establish infection of pigs by the oral route. In this study, 24 pigs were divided into four groups of six. Three of the groups were fed with a low, medium or high dose of the ASFV POL/2015/Podlaskie virus. The pigs in the fourth group served as positive controls and were inoculated intranasally, just once, using the low dose of the virus. All the pigs inoculated intranasally with ASFV succumbed to the infection, while only three of the six pigs that were fed the high dose of the virus became infected. None of the 12 pigs that were fed with either the medium or low dose of the virus became infected, despite receiving up to thirteen doses each. In two of the pigs infected by intranasal inoculation, the presence of a variant form of the ASFV genome was detected. The results obtained in this study underline that ASFV infection is more difficult to establish via the oral route when compared to the intranasal route. The high dose needed in order to establish oral infection could have implications for future strategies using baited vaccines containing infectious live-attenuated ASFV.

## 1. Introduction

African swine fever (ASF) is a hemorrhagic disease of both domestic pigs and wild boar. The disease is caused by infection with African swine fever virus (ASFV), a large dsDNA virus, the sole member of the *Asfarviridae* family [1,2]. Since 2007, a genotype II strain of ASFV has been spreading outside Africa, and thus, ASF is now reaching pandemic proportions in Europe, Asia, Oceania and the Americas [3], with a high case fatality rate in both domestic pigs and wild boar.

Although ASFV is an arbovirus, naturally transmitted by soft ticks of the genus *Ornithodoros*, it is also efficiently transmitted via direct contact between animals [1,4]. In Europe, however, most virus introductions into domestic pig herds seem to be mediated via indirect virus transmission, e.g., via pig meat or pork products, materials contaminated with carcass material or blood from infected suids or potentially via blood-feeding insects [5]. One likely route of transmission from these different materials to pigs is via oral uptake. For meat products, it has been demonstrated that ASFV can be transmitted to pigs indirectly via oral uptake of different products containing the virus [6,7,8], and infection of pigs has been demonstrated following ingestion of blood-fed stable flies [9]. Proof of efficient transmission via an environment contaminated with ASFV has not been demonstrated in experimental settings. Indeed, on the contrary, transmission from an environment contaminated with the virus has been limited or absent in such studies [10,11,12]. Studies using strict oral inoculation (i.e., oropharyngeal or via intragastric tube) with feed or insect larvae (used for food and feed) containing ASFV have also shown that it can be difficult to demonstrate infection. In one study, using doses from 1 to 10^8^ TCID_50_ in plant-based feed or liquid, the minimum infectious dose of ASFV for pigs in compound feed was reported to be 10^4^ TCID_50_, while in liquid it was found to be 1 TCID_50_ [13]. However, in another study, using commercial feed spiked with 10^4.3^ to 10^5^ TCID_50_ of the virus, pigs did not become infected even after feeding with the virus-contaminated feed on 14 consecutive days [14]. In a study using insect larvae (*Tenebrio molitor* or *Hermetia illucens*), which had fed on infectious ASFV, infection of pigs could not be demonstrated after feeding 50 virus-fed larvae to each pig. Each *T. molitor* larva had fed on 5 µL serum from an infected pig (titer 10^3.3^ TCID_50_/5 µL), while *H. illucens* had fed on feed containing a virus load of 10^5.0^ TCID_50_/g [15].

Given the currently available information, it seems as if it can be rather difficult to establish ASFV infection via the oral route. To our knowledge, except for one published study [13], ASFV dose studies have not used strict oral inoculations. For example, one dose study used intraoropharyngeal inoculation [16], while another used oronasal inoculation [17]. Therefore, we aimed to investigate the dose of a European genotype II ASFV needed to establish oral infection of pigs in our experimental settings. In such animal studies, thorough characterization of the pathogens used for inoculation is important. Therefore, we also aimed to use molecular methods to analyze the inoculation material and the viruses recovered from the infected pigs.

## 2. Materials and Methods

### 2.1. Pigs and Housing

Twenty-four male pigs, at six weeks of age, were included in this study. The pigs were Landrace x Large White and were obtained from a conventional swine herd in Catalonia, Spain. During the experiment, the pigs were housed, under high containment, at the Centre de Recerca en Sanitat Animal (IRTA-CReSA, Barcelona, Spain). On arrival at the IRTA-CReSA, the pigs were found to be healthy upon veterinary inspection. Water and a commercial diet for weaned pigs were provided ad libitum.

This study was approved by the Ethical and Animal Welfare Committee of the Generalitat de Catalunya (Autonomous Government of Catalonia; permit number: 12187). All the experimental procedures, animal care and maintenance were conducted in accordance with EU legislation on animal experimentation (EU Directive 2010/63/EU).

### 2.2. Virus and Inoculation Material

For the inoculation of the pigs, the highly virulent ASFV POL/2015/Podlaskie was used. This virus was isolated from spleen material from an ASFV-infected wild boar in Poland in 2015, essentially as described previously but with an additional passage (i.e., three passages in total) in porcine pulmonary alveolar macrophages (PPAMs) [18,19].

For the intranasal inoculation of the pigs, the third passage was diluted in phosphate-buffered saline (PBS, Thermo Fisher Scientific, Waltham, MA, USA) to a final concentration of 10^3^ TCID_50_/2 mL.

For the oral inoculation (feeding) of the pigs, the third passage virus was diluted to a final concentration of 10^3^ TCID_50_/0.5 mL, 10^4^ TCID_50_/0.5 mL and 10^5^ TCID_50_/0.5 mL, respectively, in PBS (Thermo Fisher Scientific) with 5% fetal bovine serum (FBS, Gibco, Thermo Fisher Scientific). For each dilution, ice cubes (0.5 mL, ~1 × 1 × 1 cm) were then prepared using silicone ice trays. After freezing at −80 °C, one ice cube was put into one soft cake (~50 g, diameter of cake ~2 cm) per pig. This allowed the ice cubes to thaw within the cakes prior to feeding of the pigs; however, this method retained the thawed virus suspension within the cakes.

Prior to the animal experiment, a small pilot study was performed to investigate the stability of infectious ASFV within the ice cubes using different concentrations of FBS. The pilot study was performed using the 10^3^ TCID_50_, 10^4^ TCID_50_ and 10^5^ TCID_50_ (per 0.5 mL) doses of the third passage virus in either 100% FBS, PBS with 5% FBS or PBS without FBS (Gibco, Thermo Fisher Scientific). The titers of both the freshly made virus dilutions and the thawed ice cubes of the three virus dilutions with the different concentrations of FBS were determined in primary cells as described just below. This pilot study was performed in order to ensure that the final virus dose provided to the pigs was as expected after the preparation procedures.

Back-titration of the samples from the pilot study and on the inoculums from the animal experiment (syringe with virus for intranasal inoculations, ice cubes with the three virus dilutions for oral inoculations) was carried out in PPAMs. Following 72 h of incubation at 37 °C (5% CO_2_), virus-infected cells were stained using an immunoperoxidase monolayer assay (IPMA) [18,20]. Infected (red-colored) cells were identified using a light microscope and virus titers were calculated using the method described by Reed and Muench [21].

### 2.3. Study Design

The twenty-four pigs (numbered 51–74) were randomly allocated into four groups (labelled as groups 1–4), with six pigs in each. The pens were within two high containment stable units, termed boxes 5 and 6, respectively. Pigs 51–56 (group 1) and pigs 57–62 (group 2) were housed within two pens in box 5. These pens were separated by a ~2 m high solid metal wall to prevent direct contact between the groups. Pigs 63–68 (group 3) and pigs 69–74 (group 4) were housed in two similar pens in box 6. Box 5 and box 6 were completely separated from each other, with separate air supplies, equipment, clothing, etc. Personnel showered upon exiting each of the two boxes.

After an acclimatization period of one week, the pigs (in groups 1–3) were fed different doses of ASFV POL/2015/Podlaskie orally, while pigs in group 4 were inoculated intranasally with ASFV POL/2015/Podlaskie. Specifically, three groups of six pigs were fed cakes with 10^3^ TCID_50_ (pigs 51–56, group 1), 10^4^ TCID_50_ (pigs 57–62, group 2) or 10^5^ TCID_50_ (pigs 63–68, group 3) of the virus. For the oral inoculations of the pigs in groups 1–3, within each group, two pigs were separated from the remaining four pigs in the group and these two pigs were then fed with their own individual cake containing either the low, medium or high dose of the virus. This process was then repeated for the remaining four pigs in the group, using two animals at a time.

**Figure 1:**
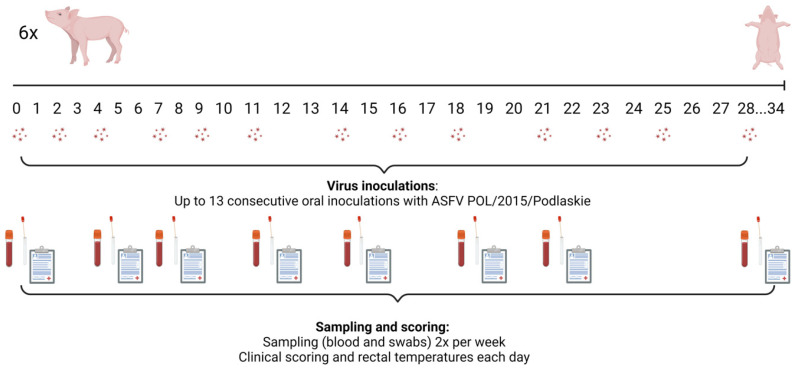
Study design for the pigs (in groups 1–3) that were administered ASFV orally in cakes. The serum tubes indicate that sampling (blood and swabs) was performed from the pigs on those days. Clinical scores and rectal temperatures were recorded daily. Created in BioRender (Toronto, ON, Canada) under the license Olesen, A. (2025) https://BioRender.com/c84u960 (accessed 10 December 2024).

In total, 13 oral inoculations were planned per group, on days 0, 2, 4, 7, 9, 11, 14, 16, 18, 21, 23, 25 and 28 (Figure 1). Pigs 69–74 (group 4) were inoculated intranasally on day 0 with 10^3^ TCID_50_ virus administered using 1 mL per nostril.

### 2.4. Clinical Examinations and Euthanasia

The clinical signs and rectal temperatures were recorded for each pig on a daily basis. Euthanasia was performed by intravascular injection of pentobarbital following deep anesthesia, either when the pigs reached the pre-determined humane endpoints or at the end of the study period.

### 2.5. Sampling

EDTA-stabilized blood (EDTA-blood), unstabilized blood samples (for serum), and nasal and oral swabs were obtained from the pigs up to twice a week, on days 0, 4, 7, 11, 14, 18, 21, 28 and at euthanasia, as depicted in Figure 1. The nasal and oral swabs were added to 1 mL of PBS (Thermo Fisher Scientific). For sampling, the pigs were restrained using a wire snare.

The collected serum, EDTA-blood and swab samples were frozen at −80 °C until further use. Prior to analysis, the swab samples were vortexed and centrifuged briefly.

### 2.6. qPCR Analysis of EDTA-Blood and Swab Samples

Nucleic acids were isolated from the EDTA-blood samples and the supernatants from the nasal and oral swabs using the MagNA Pure 96 system (Roche, Basel, Switzerland) and analyzed for the presence of ASFV DNA, essentially as described previously [18,22], using the CFX Opus Real-Time PCR System (Bio-Rad, Hercules, CA, USA). The qPCR results are presented as viral genome copy numbers per mL. The genome copy numbers were calculated based on a standard curve made from assaying a 10-fold dilution series of the pVP72 plasmid [19]. A positive result was defined as giving a threshold cycle value (Cq) at which the FAM (6-carboxy fluorescein) dye emission was above background within 42 cycles.

### 2.7. Detection of Infectious Virus in Nasal Swabs

Nasal swab samples in which ASFV DNA was readily detected (Cq value below 30) were added to PPAMs seeded into NUNC 96-well plates (Thermo Fisher Scientific) and the virus was passaged once following the addition of antibiotics and filtration of the inoculum, essentially as described previously [12]. Following inoculation, the cells were incubated at 37 °C (in 5% CO_2_) for 72 h, and the virus-infected cells were identified using IPMA, as described in Section 2.2.

### 2.8. Anti-ASFV Antibody Detection in Serum

Serum samples obtained from the pigs prior to inoculation and at euthanasia were tested for the presence of anti-ASFV antibodies using the Ingezim PPA Compac ELISA (^®^INGENASA INGEZIMPPA COMPAC K3 INGENASA, Madrid, Spain). The analysis was performed in accordance with the manufacturer’s instructions.

In addition, an in-house indirect immunoperoxidase test (IPT) was used to test for the presence of anti-ASFV antibodies. Briefly, Vero cells were inoculated with a Vero-cell-adapted ASFV POL/2015/Podlaskie and fixed following 72 h of incubation at 37 °C. Following the addition of serum samples, protein-A-conjugated horseradish peroxidase (Sigma-Aldrich, St. Louis, MO, USA) together with hydrogen peroxide and 3-amino-9-ethyl carbazole (Sigma Aldrich) were used as a chromogenic substrate.

### 2.9. Preparation of Long PCR Products from Viral DNA

DNA was extracted from the virus sample used as the inoculum (3rd passage), as in Section 2.6, and used to generate overlapping long PCR products, as previously described [23]. Briefly, amplicons derived from the inoculum sample were amplified by long PCR using AccuPrime high-fidelity DNA polymerase (Thermo Scientific, Thermo Fisher Scientific, Waltham, MA, USA). The PCR products were analyzed using the Genomic DNA ScreenTape on a 4200 TapeStation (Agilent Technologies, Santa Clara, CA, USA) and their concentrations were estimated with the Quant-iT™ 1X dsDNA broad-range kit (Invitrogen) on a FLUOstar^®^ Omega (BMG LABTECH, Mornington, VIC, Australia) instrument.

### 2.10. Variant Calling

The overlapping PCR products were pooled for the inoculum and sequenced using MiSeq (Illumina, San Diego, CA, USA) with a modified Nextera XT DNA library protocol with the MiSeq reagent kit v2 (300 cycles), resulting in 2 × 150 bp paired-end reads. The reads were trimmed using AdapterRemoval [24] by at least 30 bp at both the 5′ and 3′ ends to ensure primer removal, as well as for quality purposes (q30). Variant calling and annotation were performed using a combination of BWA-MEM, Samtools, Lo-Freq and SnpEff [25,26,27,28], as previously described [23], together with the ASFV POL/2015/Podlaskie reference sequence (MH681419.2). Variants were filtered for a minimum coverage of 50, frequency above 2%, and strand-bias Phred Score below 60.

### 2.11. Deletion Screening by PCR

The extracted DNA preparations from the EDTA-blood samples in which ASFV DNA was readily detected (Cq value below 30, corresponding to above 6.4 log_10_ genome copies/mL) were screened for the internal deletion event at pos. 6362–16,849 (10,487 bp) present within the population of viruses in the ASFV POL/Podlaskie/2015 inoculum, as described previously [23]. Briefly, the extracted DNAs were amplified by long PCR with primers spanning the deletion region at pos. 6188–17,145 (del-PCR) or primers located within this region at pos. 6708–7668 (noDel-PCR). As positive controls, we used extracted DNA from the spleen or EDTA-blood from ASFV-infected pigs, derived in previous studies [9,23], which had been shown to lack or to have the deletion, respectively. UltraPure™ DNase/RNase-Free Distilled Water was used as a negative control. The PCR products were analyzed using the Genomic DNA ScreenTape or D5000 ScreenTape on a 4200 TapeStation (Agilent Technologies) [23].

### 2.12. Nanopore Sequencing

The PCR products were cut out of agarose gels and purified using the GeneJET Gel Extraction Kit (Thermo Scientific) according to the manufacturer’s instructions, and they were then sequenced on the Nanopore (Oxford Nanopore Technologies, Oxford, UK) using a standard ligation sequencing of amplicons with native barcoding protocol for the SQK-LSK109 and native barcoding expansion kits on a R9.4.1 flow-cell. The rads were filtered for size (3200–4200 bp) using SeqKit [29] and trimmed using chopper [30] by 30 bp at both the 5′ and 3′ ends to ensure primer removal, as well as for quality purposes (q9). The trimmed reads were subsequently mapped to the ASFV POL/2015/Podlaskie reference sequence using a combination of minimap2 [31] and Samtools [26].

## 3. Results

### 3.1. Preparation and Back-Titration of the Inoculation Material

A pilot study was performed with the three different dilutions of the third passage ASFV POL/2015/Podlaskie, i.e., a low (10^3^ TCID_50_), medium (10^4^ TCID_50_) and high (10^5^ TCID_50_) dose. The dilutions were made using 100% FBS, or PBS with 5% FBS or PBS without FBS, and they were titrated in PPAM with, or without, a freeze/thaw cycle (one freeze/thaw cycle mimicked how the ice cubes used for inoculation of the pigs were handled). The pilot study indicated a stabilizing effect of both 5% FBS and 100% FBS during the freeze/thaw cycle, and this stabilizing effect was especially apparent at the low (10^3^) and medium (10^4^) doses. Hence, the final inoculation material consisted of ice cubes made from the third passage ASFV POL/2015/Podlaskie diluted in PBS with 5% FBS.

Back-titration of the inoculation material in PPAMs yielded titers of between 4.50 and 4.75 log_10_ TCID_50_ per 0.5 mL, with a mean of 4.68 log_10_ TCID_50_ for the high doses. The medium doses yielded titers from 2.51 to 3.50 log_10_ TCID_50_ per 0.5 mL, with a mean of 3.17 log_10_ TCID_50_, and the low dose titers from 1.48 to 3.25 log_10_ TCID_50_ per 0.5 mL, with a mean of 2.45 log_10_ TCID_50_. Note that the back-titrations were performed on the inoculums following two additional freeze/thaw cycles when compared to the feeding of the pigs due to shipment of the samples from Barcelona to Denmark for laboratory analysis after the animal experiment.

### 3.2. Course of Infection in Intranasally Inoculated Pigs

Following intranasal inoculation with 10^3^ TCID_50_ of ASFV, all six inoculated pigs in group 4 (pigs 69–74) presented with a high fever (rectal temperature above 41 °C) from 4–5 dpi. The clinical signs at 6 and 7 dpi included moderate to severe depression, dyspnea and ataxia. Pigs 72 and 74 were found dead upon entering the pens at 6 dpi and 7 dpi, respectively. Prior to this, a high fever had been observed in both of the pigs for two consecutive days, along with mild (pig 72) to moderate depression (pig 74) and slight dyspnea (pig 74).

**Figure 2:**
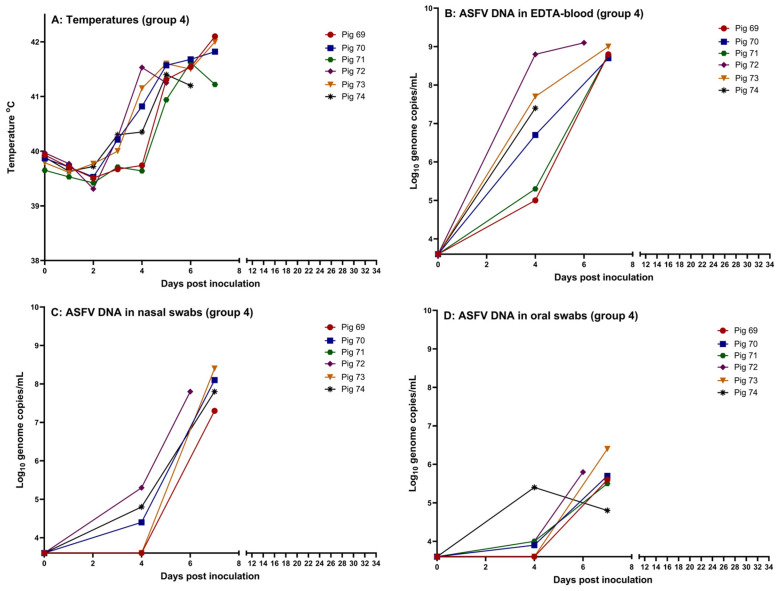
Data obtained from the intranasally inoculated pigs (pigs 69–74, group 4). (**A**) Rectal temperatures, (**B**) detection of ASFV DNA in EDTA-blood, (**C**) detection of ASFV DNA in nasal swabs, and (**D**) detection of ASFV DNA in oral swabs. The threshold for detection of ASFV DNA in the qPCR is Cq 42, which corresponds to 3.6 log_10_ genome copies/mL. Created using GraphPad Prism 9 (GraphPad Software, Boston, MA, USA).

EDTA-blood was drawn from the heart of deceased pig 72 at 6 dpi; the same, however, was not possible from pig 74 at 7 dpi. The remaining four pigs were euthanized at 7 dpi for animal welfare reasons. The rectal temperatures, for each pig, are shown in Figure 2 (panel A). Measurements of viremia, by qPCR, confirmed that high levels of viral DNA were present in EDTA-blood from the infected animals (Figure 2, panel B). ASFV DNA was also detectable in nasal and oral swabs from all six pigs, with higher levels of viral DNA in the nasal swabs compared to the oral swabs (Figure 2, panels C and D). Using the inoculation of PPAMs, infectious virus was detected in nasal swabs obtained from all six pigs at euthanasia. No anti-ASFV-specific antibodies were detected in serum from the pigs at euthanasia (see Supplementary Table S1 for a summary of the data obtained for the pigs).

### 3.3. Course of ASFV Infection in Orally Inoculated Pigs

**Figure 3:**
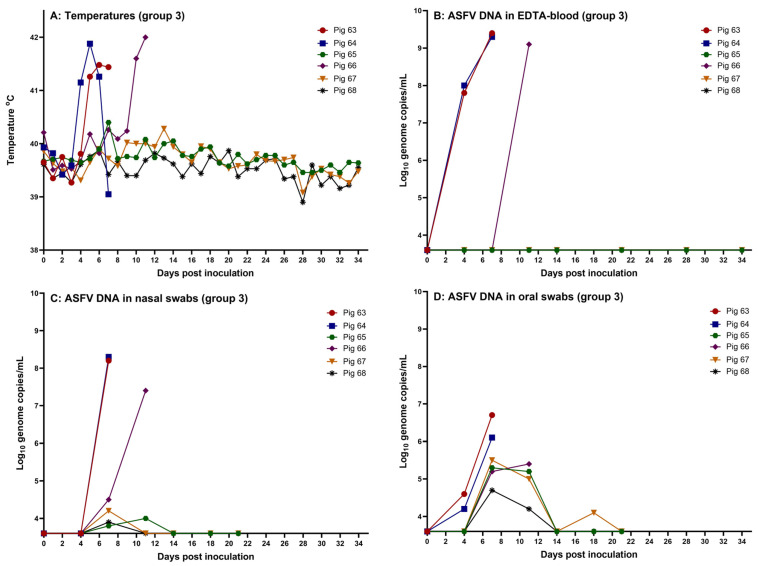
Data obtained from the pigs fed the high dose of the virus orally (pigs 63–68, group 3). (**A**) Rectal temperatures, (**B**) detection of ASFV DNA in EDTA-blood, (**C**) detection of ASFV DNA in nasal swabs, and (**D**) detection of ASFV DNA in oral swabs. The threshold for detection of ASFV DNA in the qPCR is Cq 42, which corresponds to 3.6 log_10_ genome copies/mL. Created using GraphPad Prism 9 (GraphPad Software).

Following feeding of the pigs in group 3 with the highest dose of the virus, ~10^5^ TCID_50_ (on days 0, 2 and 4), pigs 63 and 64 presented with a high fever from day 4 or 5. The clinical signs included depression, dyspnea and reddening of the skin. Two pigs reached the humane endpoints at day 7 and were euthanized. The rectal temperatures are shown in Figure 3 (panel A). As infection after feeding with the high dose was evident at this time point, no further feeding of pigs 65–68 with ASFV was performed on day 7 onwards. In addition, pig 66 had a high fever from day 10 and was euthanized on the following day when it presented with depression and dyspnea. Except for transient mild depression and diarrhea on some days, the remaining three pigs, 65, 67 and 68, despite three repeated oral inoculations, did not develop clinical signs that could indicate an infection with ASFV during the study period (Figure 3, panel B). These three pigs were euthanized on day 34, when the study was terminated. Assays for ASFV DNA in EDTA-blood provided results that were fully consistent with the clinical findings. High levels of ASFV DNA were readily detected in the blood samples obtained from pigs 63, 64 and 66, while no ASFV DNA was detected in EDTA-blood from the three remaining pigs in this group (Figure 3, panel B).

No anti-ASFV-specific antibodies were detected in the serum obtained at euthanasia from any of the six pigs within group 3 (see Supplementary Table S1).

Among the nasal and oral swabs, the highest levels of ASFV DNA were found in the samples from the three infected pigs, while lower levels of the viral DNA were found in the swabs obtained from the remaining three pigs in the pen. As also observed in the intranasally infected pigs, higher levels of viral DNA were present in the nasal swabs than in the oral swabs from the three infected pigs. However, for the three uninfected pigs (pigs 65, 67 and 68), the opposite was observed, i.e., higher levels of ASFV DNA were detected in oral swabs obtained from these pigs than in nasal swabs (Figure 3, panels C and D and Supplementary Table S2). Infectious ASFV was also detected in the nasal swabs obtained from pigs 63, 64 and 66 at euthanasia (see Supplementary Table S1).

Following feeding with either the low dose of ASFV (group 1) or the medium dose (group 2), respectively, one pig (pig 56, from group 1) that was fed the low dose presented with a high fever (and nasal mucoid discharge) on days 9 and 10. This pig was euthanized on day 11 when it appeared slightly depressed. However, no ASFV DNA was detected in the blood samples obtained from this pig (Supplementary Table S2). The remaining 11 pigs did not present with a high fever (defined as a rectal temperature above 41 °C) or with any clinical signs indicative of ASFV infection, and no ASFV DNA was detected in their blood. These pigs were euthanized on day 34, when the study was terminated. At this time point, they had each received 13 consecutive oral doses of ASFV. During the time course of the experiment, very low levels of viral DNA were detected in a few swab samples obtained from the pigs in the medium and low dose oral groups. Specifically, from the medium dose group (group 2), 3.7 and 4.5 log_10_ genome copies/mL were detected in two mouth swabs from pigs 59 and 62 on day 4, and 4.2 log_10_ genome copies/mL were present in a nasal swab from pig 61 at day 7. In the low dose oral group (group 1), 3.8 and 4.0 log_10_ genome copies/mL were detected in two oral swabs, from pig 56 (on day 7) and pig 51 (on day 11), respectively (Supplementary Table S2).

No anti-ASFV-specific antibodies were detected in the serum obtained at euthanasia from the pigs within groups 1 and 2 (see Supplementary Table S1).

### 3.4. Variant Analysis of ASFV in the Inoculum

The ASFV DNA in the inoculum was screened for the presence of single nucleotide polymorphisms (SNPs) and insertions and deletions (indels) in the MiSeq reads. All the PCR fragments had read coverage ≥ 50 for the entirety of the fragments, except for fragments 05a, 06 and 14, which lacked this degree of coverage at the 562, 124 and 405 genome positions in total, respectively. In the virus inoculum, a total of two silent SNPs were present, G33319A and A44769G, located in the MGF 505-2R and MGF 505-10R genes, respectively, at a frequency of 5.8% and 2.6%, respectively. Over 70 indels were identified, ranging in size from 1 to 3 bp; however, the majority were 1 bp in size (>68% deletions and >23% insertions), and all were located in homopolymeric regions.

### 3.5. Deletion Screening by PCR and Sequencing

**Figure 4:**
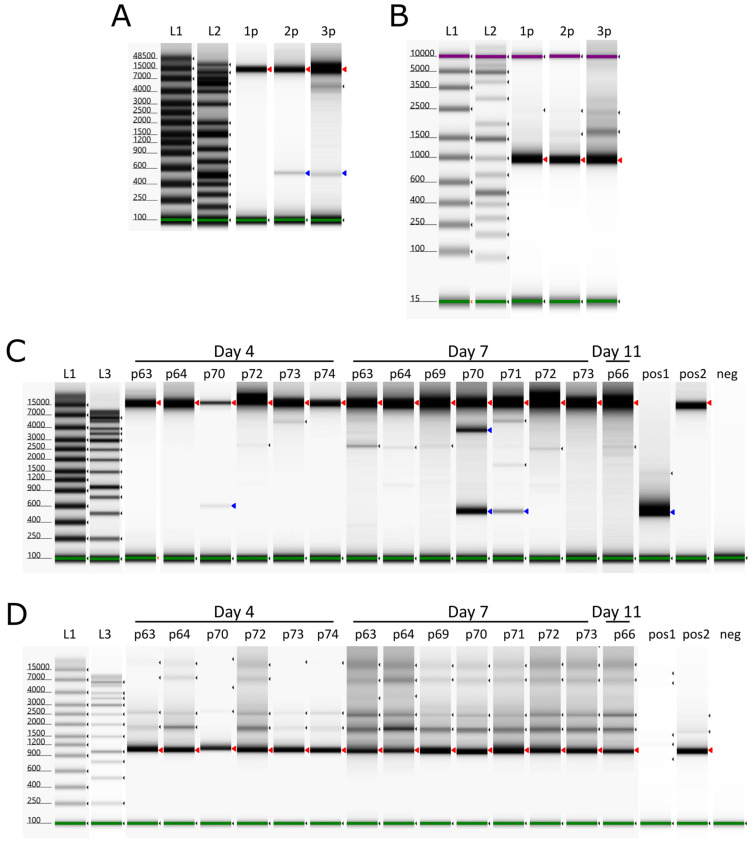
Deletion screening of ASFV POL/2015/Podlaskie inoculums (1p, 2p, 3p) together with infected pigs from group 1 (pig 63, 64, 66) and group 4 from sampling days with Cq values in the ASFV qPCR below 30 in the EDTA-blood. (**A**) PCRs with primers covering nt 6188–17,145 (del-PCR). (**B**) PCR with primers covering nt 6708–7668 (noDel-PCR). (**C**) PCR with primers covering nt 6188–17,145 (del-PCR). (**D**) PCR with primers covering nt 6708–7668 (noDel-PCR). L1: TapeStation ladder, L2: GeneRuler 1 kb plus, L3: GeneRuler 1 kb, 1p: First passage, 2p: Second passage, 3p: Third passage, p63: Pig 63 EDTA-blood, p64: Pig 64 EDTA-blood, p66: Pig 66 EDTA-blood, p69: Pig 69 EDTA-blood, p70: Pig 70 EDTA-blood, p71: Pig 71 EDTA-blood, p72: Pig 72 EDTA-blood, p73: Pig 73 EDTA-blood, p74: Pig 74 EDTA-blood, pos1: Positive control with deletion, pos2: Positive control without deletion, neg: Negative control (H_2_O). Red arrowheads indicate the wild type, whereas blue arrowheads indicate the deletion variant. Green and purple bands indicate the lower and upper molecular weight markers, respectively, whereas small black arrowheads indicate bands detected by the TapeStation Analysis software version 5.1 (Agilent Technologies).

The EDTA-blood samples and the inoculum were screened for the presence of the internal deletion event at pos. 6362–16,849 (10,487 bp), which was previously found in the second passage of the virus in PPAMs [23]. The inoculum (third passage) produced products of both ~500 bp and ~11 kb in the del-PCR, which spans the deletion site, consistent with the presence of genomes that had undergone the deletion event as well as the expected full-length PCR product, respectively (Figure 4, panel A). The product of ~1 kb in the noDel PCR, from within the deletion (Figure 4, panel B), is consistent with the ability to produce full-length (11 kb) products in the del-PCR assay. All the pigs yielded the full-length products (Figure 4 panels C and D); however, pigs 70 and 71 (from group 4) at day 7 also produced products consistent with harboring the deletion variant virus. The blood sample from pig 70 also contained shortened virus genomes at day 4; however, pig 71 was not screened in this assay on day 4 due to the low level of viral DNA present in the EDTA-blood at that time (Cq value above 30). The viral DNA from pig 70 also generated a prominent product of ~3700 bp (see Figure 4, panel C), which was extracted and sequenced using Nanopore technology, which revealed that this other deletion occurred between positions 7115 and 14,504, a total of 7389 bp; this deletion should result in a PCR product of ~3568 bp (consistent with the observed product). This deletion results in the loss of 15 complete genes; the majority are members of the MGF 110 (3L–12L) family, with a 5′ truncation of the MGF 110-2L gene and a 3′ truncation of the MGF 110-13La-13Lb, as well as ASFV G ACD 00120, 00160, 00190, 00240, MGF100-1R and 285L.

## 4. Discussion

In this study, we were able to demonstrate infection with ASFV following feeding with a high dose (10^5^ TCID_50_) of the ASFV POL/2015/Podlaskie. Oral administration of the lower doses of the same virus did not result in infection. In contrast, intranasal inoculation with the low dose (10^3^ TCID_50_) of ASFV was very efficient in establishing infection, underlining that infection with ASFV by the intranasal route is much easier to establish than infection via oral uptake. A higher efficiency of intranasal versus oral inoculation was described previously with the highly virulent ASFV-Malawi strain (genotype VIII, [32]) [16].

The relatively high dose needed for the ASFV POL/2015/Podlaskie to establish oral infection in the current study is also consistent with other studies exposing pigs to ASFV via oral uptake of feed spiked with the virus. In one study, the infection of pigs could not be established after 14 consecutive days of being fed with commercial feed spiked with serum from a pig infected with ASFV Georgia 2007/1 at levels of ~10^4^ to 10^5^ TCID_50_ [14]. In another study, using virus doses ranging from 10^3^ to 10^8^ TCID_50_, a minimum infectious dose of 10^4^ TCID_50_ was reported after one feed with compound feed spiked with spleen material from a pig infected with the ASFV Georgia 2007 [13]. In studies using oral inoculation with different insects, the feeding of 24 pigs with 50 virus-fed larvae, calculated to contain about 10^5^ TCID_50_ of the ASFV POL/2015/Podlaskie in total, did not result in infection of any of the pigs [15]. The larvae had either been fed on serum (*T. molitor*) or spleen suspension from ASFV-infected pigs (*H. illucens*). However, in an earlier study using the same virus, oral uptake of 20 blood-feeding flies fed on EDTA-blood containing infectious ASFV (calculated virus load in 20 flies was 10^5^ TCID_50_) did result in infection in 50% of the exposed pigs [9].

The reasons for the differences reported in the ability of ASFV to establish infection after the feeding of pigs, even at medium to high doses (10^4^ TCID_50_ and up), could reflect other factors than the virus dose itself. The materials used for spiking and the feeding material themselves could also impact the outcome of the virus exposures. For spiking, blood (EDTA-blood or serum), organ material (spleen) and infected-cell supernatants have each been used in various studies. It has previously been demonstrated that serum can have a stabilizing effect on ASFV [15,33], and it seems as if serum can enhance the infectivity of extracellular (but not intracellular) ASFV virions [34]. It should be noted that the studies that did report successful oral infection used spleen suspension [13], cell supernatant diluted in 5% serum (current study) or EDTA-blood [9]. On the other hand, the studies that failed to demonstrate the infection of pigs used either spleen material [15] or serum [14,15]. Hence, a clear indication of the effect of the type of spiking material used cannot be described based on the available evidence.

Another factor affecting the infection efficiency may be the feed material itself, i.e., the material and its preparation (e.g., the adsorption time of the spiking virus to the feed as also discussed previously by others [14]). These processes could have an enhancing or inhibitory effect on either the stability of the virus or the delivery of the virus dose (e.g., for establishing contact between the oropharynx and the virus). Based on the differences in the minimum infectious dose observed for the oral uptake of solid feed (10^4^ TCID_50_) versus liquid feed (1 TCID_50_) in one study, it was suggested that the liquid provided a more suitable medium for virus contact to the tonsils or other tissues where primary virus replication can occur following oronasal or intraoropharyngeal virus exposure [13]. In the current study, the soft cakes most likely provided a “sticky matrix” that allowed for prolonged exposure of the inoculation material to the lymphoid tissues in the upper gastrointestinal tract. This would include the tonsils and tissues drained by the medial retropharyngeal lymph nodes. These lymph nodes have recently been identified as key entry points for infection with ASFV in both domestic pigs and wild boar [35]. Even though the soft cakes have proven suitable for achieving oral infection, using either virus suspension in the current study or flies in a previous study [9], future studies could aim at developing more user-friendly, highly palatable and standardized delivery systems for oral exposures with ASFV (e.g., for infection and possible vaccination). From the data available so far, it seems as if a delivery system that, like the quite sticky soft cakes, allows the virus to come into prolonged contact with the upper gastrointestinal tract (e.g., using gels) could be a feasible approach.

In addition to the inoculation material used (i.e., the spiking virus and the feed), other factors can also be of importance for the outcome of this study. In the current study, a wire snare was used for restraining the pigs during sampling, which was performed prior to the inoculations of the pigs. Wire snares can result in microlesions in the oral cavity, which could be hypothesized to increase the risk of (parental) infection via these lesions, even when using lower doses of the virus. In the current study, the use of the wire snare did not, however, seem to increase the incidence of oral infection of pigs using lower doses of ASFV.

From the back-titration of the administered dose, it appears as if pigs in the low dose and medium dose groups could, unintentionally, have been given slightly lower doses than anticipated. Due to logistical reasons, the back-titrations were (non-optimally) performed after an additional two freeze/thaw cycles when compared to the time of inoculation, which could have affected the apparent level of the virus in the back-titration. If so, this effect seems to have been more pronounced in the more diluted virus suspensions (low dose and medium dose) when compared to the high dose suspension. As serum seems to have a stabilizing effect on the virus [15,33,34], a higher concentration of serum could be used for preparation of the inoculum in future studies. In the low dose and medium dose groups, the lack of infection of the pigs following 13 consecutive inoculations with virus doses from ~10^2^ to 10^4^ TCID_50_ is not consistent with the statistical model predictions from an earlier study [13]. The model applied in that study was based on infection of 40% of the pigs that were exposed to 10^4^ TCID_50_ orally, and it predicted that 10 oral exposures to 10^2^, 10^3^ or 10^4^ TCID_50_ would lead to infection of 25%, 50% and 100% of the exposed pigs, respectively. For the high dose used in the current study, 10^5^ TCID_50_, the same model was based on 44% of the pigs being exposed to 10^5^ TCID_50_ in feed becoming infected with ASFV, leading to a prediction of three exposures resulting in 75% of the exposed pigs becoming infected. In the current study, using a dose of 10^5^ TCID_50_, we found that only 33–50% of the exposed pigs were infected with ASFV following up to three doses. Further studies are required to determine the effects of multiple oral inoculations of ASFV. The apparently low ability of ASFV to establish infections via the oral route could be problematic for the development of oral vaccines to combat the disease. Currently, two intramuscular live-attenuated vaccines (LAVs) are licensed for use in domestic pigs in Vietnam [36]; however, in the European scenario, in which the virus is maintained within the wild boar population, oral vaccination using baits deployed in the field would be the most feasible approach [37]. The results obtained in the current study indicate that besides a vaccine candidate virus being effective and safe, a high dose of the vaccine virus could be needed within the bait in order to ensure successful immunization.

Due to the nature of the study design used here, it cannot be readily determined whether two or three pigs (63, 64, and 66) became infected due to feeding with the high dose of ASFV or after which of the three oral inoculations the infection was actually established. However, when comparing the time course of the infection in the three pigs to the course of infection in the intranasally inoculated pigs, it seems most likely that pigs 63 and 64 were already infected from the first inoculation at day 0. This is also in line with earlier results obtained from feeding of pigs with the same virus [9]. The delayed appearance of infection in pig 66 could either indicate that infection was established in this pig after the inoculation on day 4 or that the pig was infected via direct contact to its infected pen mates (pigs 62 and 64). Using the same virus, a four-day delay in the course of infection has previously been observed between intranasally inoculated pigs and “in contact” animals [18]. Interestingly, the lack of virus transmission to the last three pigs, pigs 65, 67 and 68, within the same pen underlines that the transmission of ASFV is not always very efficient, especially when blood is not present in the pen environment [12]. We believe that the low levels of ASFV DNA detected in the oral and nasal swabs obtained from these three pigs at day 7 indicate exposure to virus excreted from their infected pen mates, and it appears as if they were exposed primarily via the less efficient oral route. The low efficiency for establishing oral infection together with the low dose exposures have been linked to the low contagiousness of ASFV observed in field settings in Europe. This low contagiousness along with the high stability of the virus and the high case fatality rate have been linked to the persistence and spread of ASFV on the European continent [38].

The internal deletion event at pos. 6362–16,849 (10,487 bp) originally arose in the second passage in cell culture [23] and was clearly maintained in the third passage, used as inoculum in this study. It was maintained as a minority variant within the viral population of pigs 70 and 71 but does not seem to have affected pathogenicity. Pig 70 contained an additional internal deletion of 7389 bp within the same region of the genome as the 10,847 bp deletion. This smaller deletion likely occurred independently in the viral population. It could be non-essential (i.e., selected due to quicker replication of smaller genomes) or could indicate that this region of the genome is under selective pressure, suggesting a complex evolutionary landscape, with different variants competing or coexisting.

NGS revealed that, at the consensus level, the sequence of the inoculum matched the published sequence of ASFV POL/2015/Podlaskie and that only minority SNPs and 1–3 base-pair indels in homopolymeric regions were detected in the variant analysis. It is difficult to determine the veracity of such short indels in homopolymeric regions by other means, as most sequencing technologies have difficulties with these [39,40,41,42].

In conclusion, we have confirmed that infection with ASFV was not easily established following oral uptake of the virus. Only a high dose of a genotype II virus, the ASFV POL/2015/Podlaskie, was sufficient to establish infection of half of the pigs exposed by this route in our experimental setting. The high dose of ASFV needed in order to infect pigs orally could have implications for the dose needed for any future live-attenuated baited vaccine.

## Data Availability

The sequence data have been deposited as a BioProject at the NCBI (Accession number PRJNA1192983). All other necessary data are contained within the article and the Supplementary Information.

## Supplementary Materials

The following supporting information can be downloaded at https://www.mdpi.com/article/10.3390/pathogens14020119/s1, Table S1: Summary of data from pigs. Table S2: Raw data from pigs.
